# Number of Siblings and Social Capital Among Parents Rearing Schoolchildren: Results From the A-CHILD Study

**DOI:** 10.2188/jea.JE20210510

**Published:** 2023-09-05

**Authors:** Yukako Tani, Aya Isumi, Satomi Doi, Takeo Fujiwara

**Affiliations:** 1 Department of Global Health Promotion, Tokyo Medical and Dental University (TMDU), Tokyo, Japan; 2 Japan Society for the Promotion of Science, Tokyo, Japan

**Keywords:** sibling number, birth order, social capital, social relationship

## Abstract

**Background:**

Having siblings may foster sociality; however, little is known about whether sibling number determines social capital, the resources obtained through social networks. We examined the association between sibling number and social capital among Japanese parents rearing schoolchildren.

**Methods:**

We used cross-sectional data from the 2018 and 2019 Adachi Child Health Impact of Living Difficulty (A-CHILD) study, targeting all primary and junior high school students and their parents in Adachi, Tokyo, Japan (*n* = 8,082). Individual-level social capital was evaluated by assessing caregivers’ social cohesion, social support, and group affiliation. All analyses were adjusted for age and sex.

**Results:**

An inverse U-shaped association was found between sibling number and social capital. Adults who grew up with one or two, but not three or more siblings had greater social support (coefficient = 0.23; 95% confidence interval [CI], 0.06–0.40 and coefficient = 0.46; 95% CI, 0.29–0.64, respectively) than those who grew up as an only child, after covariate adjustment. Adults who grew up with two or three, but not one or four or more siblings had greater group affiliation (coefficient = 0.09; 95% CI, 0.03–0.16 and coefficient = 0.09; 95% CI, 0.01–0.18, respectively) than those who grew up as an only child, after covariate adjustment. Sibling number was not associated with social cohesion.

**Conclusion:**

Growing up with one to three siblings was associated with higher social capital in adulthood than being an only child. Having siblings may provide an opportunity to foster social capital.

## INTRODUCTION

Having siblings may foster sociality. Siblings share a family environment and spend much time together; therefore, these unique bonds provide an opportunity for children to learn about social relationships.^1^^,^^2^ A study of children in the United States showed that children with siblings have better social and interpersonal skills than children without siblings.^3^ A study of adults in the United States showed that having siblings was associated with a greater likelihood of getting married, and, once married, a lower likelihood of divorce.^4^ Data from an economics experiment investigating the effect of China’s one-child policy showed that children who had grown up during this period showed lower levels of trust, trustworthiness, and competitiveness.^5^

One measure of sociality is individual-level social capital. Although there are no universally agreed definitions of social capital, it can be described as the resources that people receive through their social networks.^6^^,^^7^ The health benefits of both individual- and community-level social capital have been demonstrated in many epidemiological studies.^6^^–^^9^ In a study of adults raising children in Japan, lack of social support was associated with a greater risk of postpartum depression.^10^ Higher parental social capital is associated with lower risk of infant physical abuse,^11^ higher levels of prosocial behaviors in children, and lower levels of behavior problems in children.^12^ However, it is not known whether growing up with multiple siblings fosters social capital in adulthood.

For adults raising children, social capital is important for both their own health and that of their children. A study of mothers of young children in Ethiopia, India, and Vietnam showed that maternal social capital moderated the association between stressful maternal life events and mental distress.^13^ A Canadian study reported that maternal social capital moderated the association between greater maternal stress and children’s emotional overeating.^14^ An Indonesian study using instrumental variable estimation showed that maternal social capital was positively associated with children’s health.^15^ Furthermore, a population-based study in Japan that used structural equation modeling showed that lower parental social capital was associated with greater parental psychological distress, which led to a higher risk of child maltreatment owing to poverty.^16^ Therefore, social capital may mitigate the negative effects of the burden of having children. However, little research has examined the determinants of social capital among adults with children.

The aim of this study was to examine the association between the number of siblings and individual-level social capital among adults raising children in Japan. Individual-level social capital has been measured from two perspectives: a network-based perspective and a social cohesion-based perspective.^17^ The former perspective has some overlap with the concept of social support. The difference between the two is that the concept of social support emphasizes resources derived from close, strong ties, whereas individual-level social capital derives more from weak ties (as well as from strong ties) and focuses on the diversity of networks.^17^ In this study, we used the latter perspective and defined social support as social capital. Hence, we measured social cohesion, social support, and group affiliations as individual-level social capital in this study.

## METHODS

### Study design and subjects

We used data from the Adachi Child Health Impact of Living Difficulty (A-CHILD) study, which was initiated in 2015 to evaluate the determinants of health among children in Adachi, Tokyo, Japan.^18^ In the present study, we used cross-sectional data collected in 2018 and 2019 on caregiver siblings. In 2018, self-report questionnaires were distributed to 6,605 pairs of children and their caregivers in elementary and junior schools (5,311 pairs in the fourth grade of 69 public elementary schools, 618 pairs in the sixth grade of nine public elementary schools, and 676 pairs in the second year of seven public junior high schools). In 2019, self-report questionnaires were distributed to 5,160 pairs in the first grade of 69 public elementary schools. Pairs of children and their caregivers completed the questionnaires at home and returned them to the school. A total of 10,221 pairs returned the questionnaires (response rate: 87%), and 9,590 pairs provided informed consent and returned all questionnaires. Data for 8,082 parents were analyzed after exclusions for caregivers other than parents (*n* = 160) and those with missing data on siblings (*n* = 1,177) or social capital (*n* = 171). The analytic sample tended to be older than the excluded sample owing to missing data on siblings and social capital (*n* = 1,348) (eTable 1). The A-CHILD protocol and use of the data for this study were approved by the ethics committee of Tokyo Medical and Dental University (No. M2016-284).

### Social capital

Social cohesion, social support, and group affiliation were evaluated as measures of individual-level social capital and assessed using the self-report questionnaire. For social cohesion, three variables (trust, cohesion, and mutual aid) were assessed using the following three questions: “Do you agree or disagree with the following statements? (1) people in your community can be trusted; (2) this community is close-knit; (3) people in your community are willing to help their neighbors.”^12^^,^^19^ Responses were rated on a five-point scale ranging from 0 for *strongly disagree* to 4 for *strongly agree*. Cronbach’s α for these three items in our sample was 0.87. The overall social cohesion score was calculated by summing the scores on the three items (range: 0–12). High scores indicated high social cohesion. We assessed social support and group affiliation using a single question adapted from the Berkman–Syme Social Network Index^20^ and modified for Japanese parents. Social support was assessed using the following question: “Do you have someone who you can consult with?”, with five response options of *none*, *1–2*, *3–4*, *5–7*, and *8 or more*. In this analysis, scores of 0, 1.5, 3.5, 6, and 8 (persons) were assigned to these categories, respectively, and the resulting variables were treated as continuous to facilitate interpretation of the results (range: 0–8). If parents did have someone who listened to their concerns and complaints, the category of person they consulted was also recorded (own parents, siblings and/or relatives, spouse or partner, parents-in-law, neighborhood friends/acquaintances, and non-neighborhood friends/acquaintances; multiple responses were allowed). Group affiliation was assessed using the following question: “Do you belong to hobby clubs, sports clubs, citizen groups, neighborhood associations, residents’ associations, etc? If so, please state how many you belong to.” To create a continuous variable to facilitate interpretation of the results, *No* responses were scored as 0 and *Yes* responses scored as the number of groups to which participants belonged.

### Siblings

The number of siblings and birth order were assessed using the self-report questionnaire. The total number of siblings reported was categorized as 0 (only child), 1, 2, 3, or ≥4; the latter category was broad because only 1.36% of participants reported having at least five siblings. For birth order, participants were classified into six categories based on their birth order and number of siblings: only child (no siblings), eldest of two siblings (firstborn with one younger sibling), eldest of three or four siblings (firstborn with two or three younger siblings), youngest of two siblings (lastborn with one older sibling), youngest of three or four siblings (lastborn with two or three older siblings), and middle of three or four siblings (middleborn with one or two older and one or two younger siblings).

### Covariates

Covariates were assessed using a self-report questionnaire. Age was divided into four categories (<35, 35–39, 40–44, and ≥45 years old). Total number of current children was divided into five categories (1, 2, 3, 4, and ≥5). Covariates with missing data were categorized as missing, and participants with missing covariate data were included in the analysis.

### Statistical analysis

First, multivariate linear regression models were used to examine the association of the number of siblings and birth order with social capital. The following sequence of models was constructed. Model 1 was adjusted for age and sex as potential confounders. Model 2 was further adjusted for total number of current children to determine if the number of siblings was associated with social capital independently of the number of children. It is likely that having more children increases the number of opportunities for parents to develop social capital. In other words, we wished to clarify the relationship between the number of siblings and social capital, excluding the effect of the number of children on the opportunities for social capital. Second, χ^2^ tests were used to assess the categories of persons participants could consult (referred to here as counselors) and differences in sibling number. Third, the association between sibling number and counselor category was evaluated using logistic regression analysis to calculate the adjusted odds ratios (ORs) with 95% confidence intervals (CIs). All analyses were conducted using STATA version 15 (Stata Statistical Software, Release 15; StataCorp LP, College Station, TX, USA).

## RESULTS

Participant characteristics are shown in Table 1. Of participants, 42% were aged <40 years and 92% were women. A total of 582 (7.2%) participants grew up as an only child, 3,662 (45%) grew up with one sibling, 2,998 (37%) grew up with two siblings, 580 (7.2%) grew up with three siblings, and 260 (3.2%) grew up with four or more siblings. Participants tended to have fewer children than the households in which they grew up. The mean social cohesion score was 7.12 (standard deviation [SD], 2.37), the mean social support score was 3.11 (SD, 1.97), and the mean social group affiliation score was 0.46 (SD, 0.75).

**Table 1.** Characteristics of participants (*n* = 8,082)

	*n*	%
Total number of siblings		
0 (only child)	582	7.2
1	3,662	45.3
2	2,998	37.1
3	580	7.2
≥4	260	3.2
Age, years		
<35	1,221	15.1
35–39	2,177	26.9
40–44	2,744	34.0
≥45	1,897	23.5
Missing	43	0.5
Sex		
Female	7,457	92.3
Male	625	7.7
Number of children		
1	1,537	19.0
2	4,008	49.6
3	2,002	24.8
4	402	5.0
≥5	103	1.3
Missing	30	0.4
	Mean	SD
Social capital		
Social cohesion (0–12)	7.12	2.37
Social support (0–8)	3.11	1.97
Group affiliation (0–7)	0.46	0.75

SD, standard deviation.

The associations between the number of siblings and social capital are shown in Table 2. An inverse U-shaped association was found between sibling number and social support and group affiliation. Participants who grew up with one or two, but not three or more siblings had greater social support (coefficient = 0.23; 95% CI, 0.06–0.40 and coefficient = 0.46; 95% CI, 0.29–0.64, respectively), compared with those who grew up as an only child, after adjusting for potential confounders (model 1). These associations were significant after adjusting for current number of children (model 2). Participants who grew up with two or three, but not one or four or more siblings had greater group affiliation (coefficient = 0.09; 95% CI, 0.03–0.16 and coefficient = 0.09; 95% CI, 0.01–0.18, respectively), compared with those who grew up as an only child, after adjusting for potential confounders (model 1). These associations were attenuated after adjusting for current number of children (model 2). Number of siblings was not associated with social cohesion (all *P* > 0.2). The same results were obtained when the data analyses included only women (data not shown).

**Table 2.** Results of regression analyses of social capital according to total sibling number among Japanese parents

			Model 1	Model 2
		Mean (SD)	coefficient (95% CI)	coefficient (95% CI)
Social cohesion (0–12)				
Total number of siblings	0 (only child)	7.12 (2.55)	referent	referent
	1	7.10 (2.30)	−0.07 (−0.2 to 0.14)	−0.09 (−0.30 to 0.12)
	2	7.17 (2.38)	0.06 (−0.15 to 0.27)	0.02 (−0.18 to 0.23)
	3	7.10 (2.48)	0.02 (−0.25 to 0.29)	−0.01 (−0.28 to 0.26)
	≥4	6.82 (2.65)	−0.29 (−0.63 to 0.06)	−0.32 (−0.66 to 0.03)
Social support (0–8)				
Total number of siblings	0 (only child)	2.83 (1.89)	referent	referent
	1	3.05 (1.94)	**0.23 (0.06**–**0.40)**	**0.22 (0.05**–**0.40)**
	2	3.29 (2.04)	**0.46 (0.29**–**0.64)**	**0.46 (0.28**–**0.63)**
	3	2.99 (1.86)	0.16 (−0.06 to 0.39)	0.17 (−0.05 to 0.40)
	≥4	2.86 (1.89)	0.05 (−0.24 to 0.34)	0.08 (−0.21 to 0.36)
Group affiliation (0–7)				
Total number of siblings	0 (only child)	0.39 (0.64)	referent	Referent
	1	0.46 (0.76)	0.05 (−0.01 to 0.12)	0.04 (−0.02 to 0.11)
	2	0.48 (0.77)	**0.09 (0.03**–**0.16)**	**0.07 (0.008**–**0.14)**
	3	0.47 (0.75)	**0.09 (0.01**–**0.18)**	0.07 (−0.01 to 0.16)
	≥4	0.41 (0.73)	0.02 (−0.09 to 0.13)	−0.01 (−0.12 to 0.10)

CI, confidence interval; SD, standard deviation.

Model 1: Adjusted for age and sex.

Model 2: Model 1 + adjusted for number of children.

The associations of birth order and number of siblings with social capital are shown in Table 3. Regardless of birth order, participants who grew up with two or three siblings had greater social support (coefficient = 0.33; 95% CI, 0.11–0.55 for eldest, coefficient = 0.44; 95% CI, 0.25–0.62 for middle, and coefficient = 0.36; 95% CI, 0.16–0.55 for youngest of three or four siblings), compared with those who grew up as an only child, after adjusting for potential confounders (model 1). Of participants who grew up with one sibling, only firstborns reported greater social support (coefficient = 0.38; 95% CI, 0.18–0.57) than those who grew up as an only child (model 1). These associations were significant after adjusting for current number of children (model 2). For group affiliation, participants who had younger sibling(s) had greater group affiliation (coefficient = 0.15; 95% CI, 0.07–0.22 for eldest of two siblings, coefficient = 0.13; 95% CI, 0.05–0.22 for eldest of three or four siblings, and coefficient = 0.09; 95% CI, 0.02–0.16 for middle of three or four siblings), compared with those who grew up as an only child, after adjusting for potential confounders (model 1). These associations were attenuated after adjusting for current number of children (model 2). Birth order was not associated with social cohesion.

**Table 3.** Results of regression analyses of social capital according to birth order and sibling number among Japanese parents

			Model 1	Model 2
		Mean (SD)	coefficient (95% CI)	coefficient (95% CI)
Social cohesion (0–12)				
Birth order	Only child	7.12 (2.55)	referent	referent
	Eldest of 2 siblings	7.21 (2.35)	0.02 (−0.22 to 0.25)	−0.003 (−0.24 to 0.23)
	Eldest of 3/4 siblings	7.32 (2.43)	0.13 (−0.14 to 0.40)	0.09 (−0.18 to 0.36)
	Youngest of 2 siblings	7.04 (2.26)	−0.11 (−0.33 to 0.10)	−0.13 (−0.34 to 0.08)
	Youngest of 3/4 siblings	7.19 (2.43)	0.04 (−0.19 to 0.27)	0.002 (−0.23 to 0.23)
	Middle of 3/4 siblings	7.08 (2.36)	−0.01 (−0.23 to 0.21)	−0.03 (−0.25 to 0.18)
Social support (0–8)				
Birth order	Only child	2.83 (1.89)	referent	referent
	Eldest of 2 siblings	3.17 (1.98)	**0.38 (0.18**–**0.57)**	**0.37 (0.18**–**0.57)**
	Eldest of 3/4 siblings	3.15 (2.03)	**0.33 (0.11**–**0.55)**	**0.33 (0.10**–**0.55)**
	Youngest of 2 siblings	2.99 (1.91)	0.16 (−0.02 to 0.33)	0.15 (−0.03 to 0.33)
	Youngest of 3/4 siblings	3.23 (1.96)	**0.36 (0.16**–**0.55)**	**0.35 (0.16**–**0.54)**
	Middle of 3/4 siblings	3.28 (2.05)	**0.44 (0.25**–**0.62)**	**0.44 (0.25**–**0.62)**
Group affiliation (0–7)				
Birth order	Only child	0.39 (0.64)	referent	referent
	Eldest of 2 siblings	0.57 (0.86)	**0.15 (0.07**–**0.22)**	**0.13 (0.06**–**0.21)**
	Eldest of 3/4 siblings	0.54 (0.84)	**0.13 (0.05**–**0.22)**	**0.11 (0.02**–**0.19)**
	Youngest of 2 siblings	0.41 (0.71)	0.01 (−0.06 to 0.08)	0.002 (−0.07 to 0.07)
	Youngest of 3/4 siblings	0.46 (0.72)	0.06 (−0.02 to 0.13)	0.04 (−0.04 to 0.11)
	Middle of 3/4 siblings	0.48 (0.76)	**0.09 (0.02**–**0.16)**	**0.08 (0.01**–**0.15)**

CI, confidence interval; SD, standard deviation.

Model 1: Adjusted for age and sex.

Model 2: Model 1 + adjusted for number of children.

Counselor categories for parents receiving social support from at least one person are shown in eTable 2. The most common types of counselor were spouses and own parents (76% for spouse and 71% for own parents, respectively). This was followed by 48% and 42% for neighborhood friends/acquaintances and siblings and/or relatives, respectively. There was a difference in counselor category (except parents-in-law and work colleagues) according to sibling number. The association between sibling number and counselor category after adjusting for potential confounders is shown in Table 4. An inverse U-shaped association was found between sibling number and whether or not participants could consult their own parents. Compared with participants who grew up as an only child, those who grew up with one or two siblings were more likely to choose their parents to consult, but those who grew up with four or more siblings were less likely to choose their parents to consult (OR 1.48; 95% CI, 1.22–1.79 for one sibling, OR 1.35; 95% CI, 1.11–1.64 for two siblings, and OR 0.53; 95% CI, 0.39–0.72 for four or more siblings). However, as the number of siblings increased, parents chose to consult siblings and/or relatives. Compared with participants who grew up as an only child, those who grew up with four or more siblings were less likely to choose spouses and neighborhood friends to consult (OR 0.64; 95% CI, 0.46–0.89, and OR 0.54; 95% CI, 0.39–0.74, respectively). Compared with participants who grew up as an only child, those who grew up with siblings were less likely to choose non-neighborhood friends to consult (OR 0.83; 95% CI, 0.69–0.99 for one sibling, OR 0.81; 95% CI, 0.67–0.97 for two siblings, OR 0.66; 95% CI, 0.52–0.85 for three siblings, and OR 0.65; 95% CI, 0.46–0.90 for four or more siblings).

**Table 4.** Adjusted odds ratios of counselor type according to total sibling number among Japanese parents receiving social support from at least one person (*n* = 7,780)

		Type of counselor				
		Own parents	Siblings and/or relatives	Spouse or partner	Neighborhood friends/acquaintances	Non-neighborhood friends/acquaintances
		OR (95% CI)	OR (95% CI)	OR (95% CI)	OR (95% CI)	OR (95% CI)
Total number of siblings	0 (only child)	referent	referent	referent	referent	referent
	1	**1.48 (1.22–1.79)**	**4.53 (3.47–5.92)**	1.04 (0.85–1.29)	0.91 (0.76–1.09)	**0.83 (0.69–0.99)**
	2	**1.35 (1.11–1.64)**	**6.90 (5.28–9.04)**	1.14 (0.92–1.41)	0.99 (0.82–1.18)	**0.81 (0.67–0.97)**
	3	0.90 (0.70–1.16)	**9.48 (6.97–12.90)**	0.93 (0.71–1.22)	0.83 (0.65–1.05)	**0.66 (0.52–0.85)**
	≥4	**0.53 (0.39–0.72)**	**13.50 (9.35–19.50)**	**0.64 (0.46–0.89)**	**0.54 (0.39–0.74)**	**0.65 (0.46–0.90)**

CI, confidence interval; OR, odds ratio.

Model: Adjusted for age and sex.

## DISCUSSION

To our knowledge, this is the first study to examine the association between the number of siblings and individual-level social capital among parents raising children. We found an inverse U-shaped association between sibling number and social capital, and optimal number of siblings differed according to the type of social capital. Compared with parents who grew up as an only child, those who grew up with one to three siblings had higher individual-level social capital (eg, social support and group affiliation); however, this effect was not found for parents who grew up with four or more siblings.

We found an inverse U-shaped association between sibling number and social support, and the optimal number of siblings was one or two. This is in accord with a study conducted in the United States that found that children with one to two siblings, rather than three or more siblings, had better interpersonal skills than those without siblings.^3^ These results suggest that interpersonal skills are better in individuals with one or two siblings but that this benefit declines with three or more siblings. This may be because individuals who grow up with one to two siblings may have a good relationship with their own parents and have greater opportunities to learn social skills from parents at home. We found that participants who grew up as an only child and those who grew up with three or more siblings were less likely to choose their own parents to consult compared with those who grew up with one to two siblings. In one-child families, both positive and negative parental influences (eg, pressure) may be focused on the child. We found that participants who grew up as an only child tended to choose non-neighborhood friends to consult, suggesting that these individuals may choose to rely on friends rather than parents, regardless of where they live. Additionally, it may be important that only children do not have the opportunity to cultivate interpersonal skills with their siblings. In families with many children, parental resources may be insufficient, which is consistent with the dominant theory used to explain the consequences of sibling size, “resource dilution.”^21^^,^^22^ Resource dilution theory posits that parental resources such as time, energy, and money are diluted among a large number of siblings; therefore, children who grow up with many siblings have fewer advantages than those with fewer or no siblings.^21^^,^^23^ We found that participants who grew up with four or more siblings were less likely to choose spouses and neighborhood friends to consult. Therefore, those who grew up with many siblings may have fewer social skills with which to develop supportive relationships in adulthood.

The associations between the number of siblings and individual-level social capital were attenuated after adjusting for current number of children, especially in relation to group affiliation. We confirmed that the larger the number of siblings, the larger the number of children. Therefore, having more children may increase the opportunities for parents to participate in some groups.

We found that the youngest of three or four siblings showed greater social support, although the youngest of two siblings did not. This may be because as the number of siblings increased, parents had the option of consulting more than one sibling. Among participants who grew up with one sibling, only firstborns reported greater social support. This may be explained by differences in counselor category. We found that compared with firstborns, later-born participants were less likely to choose their parents to consult (data not shown).

We found that participants who grew up with two to three siblings and those with younger sibling(s) had high group affiliation. Those who grew up with two to three siblings and had younger siblings may have experienced many opportunities for leadership since childhood, so they were confident in joining social groups outside the home, or had been asked by others to join such groups. Additional research is needed to investigate the type of group affiliation and the motives for participation in social groups.

We found that sibling number was not associated with social cohesion. Whereas social support and group affiliation comprise the structural or functional aspects of social capital, social cohesion is a cognitive aspect of social capital and may have different determinants from other aspects of social capital. Several studies have shown that neighborhood safety is associated with social cohesion.^24^^,^^25^ A study of older people in Japan showed that those who participated in community intervention programs called *Kayoino-Ba* had increased cognitive social capital.^26^ In our sample, we found that parents whose children participated in community events, such as festivals, Christmas parties, and disaster drills, had greater social cohesion than those whose children did not participate in community events (data not shown). Therefore, macrolevel environments may contribute more to building social cohesion than microlevel environments, such as the family.

This study had several limitations. First, we could not assess the effect of the sex of siblings. A study of Japanese children showed that the sex of siblings affected the association between sibling number and mental health.^27^ However, whether this limitation led to underestimation or overestimation of the effect in the present study is unclear because the relationship between sibling sex and social capital is unknown. Second, we lacked information on potential confounders, such as socioeconomic status and health status during childhood. However, the relationship between socioeconomic status and the number of siblings may not be linear in developed countries. In our sample, the relationship between household income and current number of children was a loose U-shaped association. Therefore, it is unclear whether this limitation led to underestimation or overestimation of the effect. Poor health status during childhood may be associated with having fewer younger siblings and, therefore, with less development of social capital. Future studies need to include appropriate measures of childhood health status. Third, social support and group affiliation were assessed using a single item, which has not been tested for reliability and validity with Japanese parents. Future studies should use more detailed questions to assess which aspects of social support and group affiliation are associated with number of siblings. Finally, the sample was limited to adults with children attending public schools in one city in Tokyo, so the generalizability of the findings to parents with children attending private schools or residing in other areas may not be high.

This study has produced novel findings regarding the association between number of siblings and individual-level social capital among parents with children. We found an inverse U-shaped association between sibling number and social capital. It may be important for an only child to spend time with children of different ages, such as in mixed-age childcare,^28^ and for a child with four or more siblings to spend time with adults other than parents. Considering the health benefits of social capital, future studies should investigate the mechanism underlying the association between sibling number and social capital and confirm this association among other populations and regions.

## SUPPLEMENTARY MATERIAL

eTable 1. Characteristics of study sample (n = 8,082) and excluded sample owing to missing data on siblings and social capital (n = 1,348)

eTable 2. Type of counselor for Japanese parents receiving social support from at least one person (n = 7,780)
