# Supplementary material for: Number of Siblings and Social Capital Among Parents Rearing Schoolchildren: Results From the A-CHILD Study
Source: J Epidemiol. 2023 Sep 5;33(9):478–83. doi: 10.2188/jea.JE20210510 (PMC10409528; doi:10.2188/jea.JE20210510)
Supplement: Supplementary file 1 [file je-33-478-s001.pdf]

eTable 1. Characteristics of study sample (n=8,082) and excluded sample owing to missing data on siblings and social capital (n=1,348)

	Study sample (n=8,082)		Excluded sample (n=1,348)		*P*-value[a]
	n	%	n	%	
Age, years					
<35	1,221	15.1	243	18.0	<0.001
35–39	2,177	26.9	359	26.6	
40–44	2,744	34.0	404	30.0	
≥45	1,897	23.5	180	13.4	
Missing	43	0.5	162	12.0	
Sex					
Female	7,457	92.3	1,239	91.9	0.65
Male	625	7.7	109	8.1	
Household income, million yen					
<3.00	535	6.6	37	2.7	<0.001
3.00–5.99	1,552	19.2	39	2.9	
6.00–9.99	1,738	21.5	31	2.3	
≥10.0	575	7.1	19	1.4	
Missing	3,682	45.6	1,222	90.7	
Medical history					
Cardiovascular disease	132	1.6	35	2.6	0.01
Asthma	763	9.4	133	9.9	0.62
Diabetes	104	1.3	7	0.5	0.02
Psychiatric disease	419	5.2	64	4.7	0.50
Number of children					
1	1,537	19.0	306	22.7	0.003
2	4,008	49.6	676	50.1	
3	2,002	24.8	291	21.6	
4	402	5.0	49	3.6	
≥5	103	1.3	19	1.4	
Missing	30	0.4	7	0.5	

[a]Differences were tested using Pearson’s chi-square test.

eTable 2. Type of counselor for Japanese parents receiving social support from at least one person (n=7,780)

	Total		Total number of siblings					P-value[a]
			0 (only child)	1	2	3	≥4	
	n	%	%	%	%	%	%	
Type of counselor								
Own parents	5,548	71.3	66.6	73.7	72.5	63.9	50.4	<0.0001
Siblings and/or relatives	3,232	41.5	11.7	37.3	47.8	55.7	63.8	<0.0001
Spouse or partner	5,922	76.1	75.4	76.4	77.4	73.2	65.4	<0.0001
Parents-in-law	1,404	18.0	16.7	18.1	18.7	18.4	12.2	0.13
Neighborhood friends/acquaintances	3,693	47.5	49.4	47.3	49.1	44.5	33.7	<0.0001
Non-neighborhood friends/acquaintances	2,535	32.6	37.3	32.9	32.5	28.4	27.6	0.01
Work colleagues	1,470	18.9	19.4	18.2	19.6	20.4	16.3	0.40

[a]Differences were tested using Pearson's chi-square test.
